# Supplementary material for: The application of cell-free DNA methylation patterns in critical illnesses: A protocol paper
Source: PLoS One. 2025 May 12;20(5):e0321626. doi: 10.1371/journal.pone.0321626 (PMC12068884; doi:10.1371/journal.pone.0321626)
Supplement: S1 File. — The application of cell-free DNA methylation patterns in critical illnesses protocol. (PDF) [file pone.0321626.s001.pdf]

Title: “The application of cell-free DNA methylation patterns in critical illnesses protocol.”

1. Study design
    1.1. This is a protocol paper describing the use of cell-free DNA as a biomarker in a swine model of cardiac arrest using carbon monoxide as a therapy.
    1.2. This was built off a recent publication in PLOS ONE entitled, “Carbon monoxide as a cellular protective agent in a swine model of cardiac arrest protocol. PLoS One. 2024 May 15;
    1.3. In a sample of this ongoing large animal study to demonstrate our protocol, all procedures were approved by the Institutional Animal Care and Use Committee at the Children’s Hospital of Philadelphia (CHOP) and performed in accordance with the National Institutes of Health Guide for the Care and Use of Laboratory Animals (Protocol 1443). Yorkshire pigs (weight ~ 10 kg and approximately 1-2 months of chronological age), equal numbers of each sex, were used. 19(5):e0302653.”

2. Analysis Plan

You may describe one or more confirmatory analysis in this preregistration. Please remember that all analyses specified below must be reported in the final article, and any additional analyses must be noted as exploratory or hypothesis generating.

A confirmatory analysis plan must state up front which variables are predictors (independent) and which are the outcomes (dependent), otherwise it is an exploratory analysis. You are allowed to describe any exploratory work here, but a clear confirmatory analysis is required.

3. Statistical models (required)**.** The EdgeR package in Seqmonk was applied to DMR analysis. The Benjamini-Hochberg procedure was carried out to adjust p-values for multiple comparisons.
