# Supplementary material for: The application of cell-free DNA methylation patterns in critical illnesses: A protocol paper
Source: PLoS One. 2025 May 12;20(5):e0321626. doi: 10.1371/journal.pone.0321626 (PMC12068884; doi:10.1371/journal.pone.0321626)
Supplement: Attachment — Submitted filename: Response to Reviewers.docx [file pone.0321626.s002.docx]

PONE-D-24-42835

The application of cell-free DNA methylation patterns in critical illnesses protocol

PLOS ONE

Dear PLOS ONE,

Thank-you for the reviewer comments to improve this paper. We have submitted a revised manuscript that should address all of the feedback. Please let us know if you require any additional information and/or changes.

We have included the following for this submission:

- [ ] A rebuttal letter that responds to each point raised by the academic editor and reviewer(s). You should upload this letter as a separate file labeled 'Response to Reviewers'.
- [ ] A marked-up copy of your manuscript that highlights changes made to the original version. You should upload this as a separate file labeled 'Revised Manuscript with Track Changes'.
- [ ] An unmarked version of your revised paper without tracked changes. You should upload this as a separate file labeled 'Manuscript'.

For this submission we also have addressed the following and/or respond to the following:

[x] We note you have not yet provided a [protocols.io](http://protocols.io/) PDF version of your protocol and/or a [protocols.io](http://protocols.io/) DOI. When you submit your revision, please provide a PDF version of your protocol as generated by [protocols.io](http://protocols.io/) (the file will have the [protocols.io](http://protocols.io/) logo in the upper right corner of the first page) as a Supporting Information file. The filename should be S1_file.pdf, and you should enter “S1 File” into the Description field. Any additional protocols should be numbered S2, S3, and so on. Please also follow the instructions for Supporting Information captions [<https://journals.plos.org/plosone/s/supporting-information#loc-captions>]. The title in the caption should read: “Step-by-step protocol, also available on protocols.io.”

Please assign your protocol a [protocols.io](http://protocols.io/) DOI, if you have not already done so, and include the following line in the Materials and Methods section of your manuscript: “The protocol described in this peer-reviewed article is published on [protocols.io](http://protocols.io/) ([https://dx.doi.org/10.17504/protocols.io.[...]](https://dx.doi.org/10.17504/protocols.io.%255B...%255D)) and is included for printing purposes as S1 File.” You should also supply the DOI in the Protocols.io DOI field of the submission form when you submit your revision.

If you have not yet uploaded your protocol to [protocols.io](http://protocols.io/), you are invited to use the platform’s protocol entry service [<https://www.protocols.io/we-enter-protocols>] for doing so, at no charge. Through this service, the team at [protocols.io](http://protocols.io/) will enter your protocol for you and format it in a way that takes advantage of the platform’s features. When submitting your protocol to the protocol entry service please include the customer code PLOS2022 in the Note field and indicate that your protocol is associated with a PLOS ONE Lab Protocol Submission. You should also include the title and manuscript number of your PLOS ONE submission.

**RESPONSE: Statistical analyses. The EdgeR package in Seqmonk was applied to DMR analysis. The Benjamini-Hochberg procedure was carried out to adjust p-values for multiple comparisons The protocol described in this peer-reviewed article is published on protocols.io.: (https://www.protocols.io/view/the-application-of-cell-free-dna-methylation-patte-dwau7aew)**

[x] Please include a link to the [protocols.io](http://protocols.io/) entry in your Methods section.

**RESPONSE:**

[x] We noticed you have some minor occurrence of overlapping text with the following previous publication(s), which needs to be addressed:

Cell-Free DNA as a Biomarker in a Rodent Model of Chlorpyrifos Poisoning Causing Mitochondrial Dysfunction - <https://doi.org/10.1007/s13181-023-00956-0>

 In your revision ensure you cite all your sources (including your own works), and quote or rephrase any duplicated text outside the methods section. Further consideration is dependent on these concerns being addressed. In addition, the methods of this paper

**RESPONSE: We revised the introduction section to ensure there is no overlap with our cited publication listed above. Also, for the methods we made minor revisions more specific to in swine model as opposed to the use of cfDNA in rodents which is what the paper above describes. The methods from the paper below is focused in the use in our swine model and DNA methylation pattern which is not present in our previous publication cited above.**

**To illustrate our protocol levering our established swine model of cardiac arrest with the use of CO as a therapy,** blood samples were then collected following a standard protocol using cfDNA/cfRNA Preservative Norgen tubes (Norgen biotek, Canada). Briefly, a total of 20 ml of whole blood placement into the Norgen tubes, each tube was then gently inverted 5 times to allow gentle mixture of the preservative and the blood samples. Samples were then centrifuged at 1,600 x g for 10 min at 4°C to obtain plasma on the upper layer. For cfDNA, the plasma was then centrifuged at 16,000 x g at RT to remove cell debris. The supernatant was then stored at -80°C before cfDNA extraction. cfDNA extraction was then performed with the NextPrep-Mag cfDNA isolation kit (PerkinElmer, MA), following the manufacturer’s protocol. CfDNA quantification was performed using Qubit 1x dsDNA HS assay kit with the Qubit 4 fluorometer (Thermo Fisher Scientific). Swine cortex (PG-212) and blood (PG-705) genomic DNA (gDNA) samples were acquired from Zyagen as reference genomes. Samples were bisulfite converted using EZ DNA methylation-lightning kit (Zymo Research) before sequencing and data processing.^12,13^

Libraries for the WGBS were created from the bisulfite-converted samples using the NEBNext Enzymatic Methyl-seq Kit (New England Biolabs) according to the manufacturer’s protocol. Libraries were quantified with the Qubit 1x dsDNA high sensitivity assay kit and the Qubit 4 fluorometer. The WGBS was conducted on the NovaSeq 6000 platform (Illumina) producing 2 x 150 paired end reads at 30 x coverage. Sequencing reads were analyzed as follows: quality checked, trimmming, and aligned to the pig reference genome assembly, respectively. Bismark methylation extraction was applied to extract the methylation call for CpGs in each sample.^14^

**Differential Methylation Analysis.** We used the Seqmonk analytic workflow to identify DMRs among tissue types (<https://www.bioinformatics.babraham.ac.uk/projects/seqmonk/>). All DMRs were based on a genomic window size of 25 CpGs. Methylation values were quantitated by the “bisulphite methylation over features” pipeline in Seqmonk. The following criteria were applied for the selection of the following DMRs. Brain-specific hypermethylated DMRs were defined when the amount of methylation in cortex exceeded that of blood by more than 50 (differential (diff) methylation >50), and hypomethylated DMRs were defined as diff methylation < 50, with an FDR < 0.00005.

**Statistical analyses.** The EdgeR package in Seqmonk was applied to DMR analysis. The Benjamini-Hochberg procedure was carried out to adjust p-values for multiple comparisons.

[x] Please note that PLOS ONE has specific guidelines on code sharing for submissions in which author-generated code underpins the findings in the manuscript. In these cases, we expect all author-generated code to be made available without restrictions upon publication of the work. Please review our guidelines at <https://journals.plos.org/plosone/s/materials-and-software-sharing#loc-sharing-code> and ensure that your code is shared in a way that follows best practice and facilitates reproducibility and reuse.

**RESPONSE:** We did not have any code involved with this study did upload our raw data that is publicly available on Mendeley Data under the same title of this manuscript.

[x] We note that the grant information you provided in the ‘Funding Information’ and ‘Financial Disclosure’ sections do not match.

When you resubmit, please ensure that you provide the correct grant numbers for the awards you received for your study in the ‘Funding Information’ section.

**RESPONSE: I put in my funding (R01HL166592) under both Financial Disclosure and Funding information.**

[x] We note that your Data Availability Statement is currently as follows: All relevant data are within the manuscript and its Supporting Information files.

Please confirm at this time whether or not your submission contains all raw data required to replicate the results of your study. Authors must share the “minimal data set” for their submission. PLOS defines the minimal data set to consist of the data required to replicate all study findings reported in the article, as well as related metadata and methods (<https://journals.plos.org/plosone/s/data-availability#loc-minimal-data-set-definition>).

For example, authors should submit the following data:

- The values behind the means, standard deviations and other measures reported;

- The values used to build graphs;

- The points extracted from images for analysis.

Authors do not need to submit their entire data set if only a portion of the data was used in the reported study.

If your submission does not contain these data, please either upload them as Supporting Information files or deposit them to a stable, public repository and provide us with the relevant URLs, DOIs, or accession numbers. For a list of recommended repositories, please see <https://journals.plos.org/plosone/s/recommended-repositories>.

If there are ethical or legal restrictions on sharing a de-identified data set, please explain them in detail (e.g., data contain potentially sensitive information, data are owned by a third-party organization, etc.) and who has imposed them (e.g., an ethics committee). Please also provide contact information for a data access committee, ethics committee, or other institutional body to which data requests may be sent. If data are owned by a third party, please indicate how others may request data access.

**RESPONSE: All our raw data is on Mendeley Data under the same title of this manuscript that is publicly available.**

[x] Please include a separate caption for each figure in your manuscript.

**RESPONSE: We now have separate caption for each figure**

[x] Please include captions for your Supporting Information files at the end of your manuscript, and update any in-text citations to match accordingly. Please see our Supporting Information guidelines for more information: <http://journals.plos.org/plosone/s/supporting-information>.

**RESPONSE: This protocol paper does not contain and supporting information files.**

[x] Please review your reference list to ensure that it is complete and correct. If you have cited papers that have been retracted, please include the rationale for doing so in the manuscript text, or remove these references and replace them with relevant current references. Any changes to the reference list should be mentioned in the rebuttal letter that accompanies your revised manuscript. If you need to cite a retracted article, indicate the article’s retracted status in the References list and also include a citation and full reference for the retraction notice.

**RESPONSE: Our reference is complete and correct upon second review.**

**Response to reviewer comments:**

**Reviewer #1: In this manuscript, the authors propose a procedure using samples in a swine model of cardiac arrest where carbon monoxide is being used as a therapeutic to demonstrate the method and feasibility to obtain plasma cfDNA methylation patterns to help identify tissue origin with potential application in critical care medicine. In general, the manuscript is well written, focused and proposes new role for liquid markers.
However, the authors should address some specific points:**

1. The authors state that Yorkshire pigs (weight ~ 10 kg and 95 approximately 1-2 months of chronological age), equal numbers of each sex, were used. The number of animals and their allocation in groups/subgroups should be stated. Moreover, a short table showing how the animals were divided and in which groups would be advisable.

**RESPONSE:** **This was a protocol manuscript to described for a future upcoming study and we performed on two animals for each group to highlight our methods. We revised the results section to include this information to highlight this fact**

Result section under cell-free DNA section:

*To examine whether CO treatment makes a difference to animals suffering from cardiac arrest, we profiled the cfDNA methylome in Sham and CO groups using two animals in each group to illustrate our methods*

2. I could not access more extensive data that is a specific requirement of the journal. Please, provide these as an additional/complimentary data.

**RESPONSE: All our raw data is now publicly available on Mendeley Data under the same title of this manuscript**

**Reviewer #2: This manuscript by Kao et al. entitled “The application of cell-free DNA methylation patterns in critical illnesses protocol” aims to provide insights into the utility of cfDNA with accompanying methylome analysis as a tissue-specific biomarker in critical illness. Overall, this proof of concept was successfully achieved, however there are some clarifications that are required:**

Major comments:
1) Please consider rephrasing your definition of Brain-specific DMRs. The definition becomes clear when viewing Fig B. However, when reviewing materials and methods, it suggests that a hypomethylated DMR is defined as a methylation difference of 0-49% in brain as compared to blood (page 16, line 163). This could be simplified to state “Brain-specific DMRs were defined as those regions in which methylation in the cortex differed from methylation in blood by more than 50% (differential (diff) methylation >50%). Positive diff methylation indicates relative hypermethylation of the brain-specific DMR relative to blood, whereas negative diff methylation indicates relative hypomethylation.”

**RESPONSE:** **We revised the Diferentual Methylation Analysis section as recommended above to address the reviewer comments.**

Methods under Differential Methylation Analysis section

*We used the Seqmonk analytic workflow to identify DMRs among tissue types (https://www.bioinformatics.babraham.ac.uk/projects/seqmonk/). All DMRs were based on a genomic window size of 25 CpGs. Methylation values were quantitated by the “bisulphite methylation over features” pipeline in Seqmonk. The following criteria were applied for the selection of the following DMRs. Brain-specific DMRs were defined as those regions in which methylation in the cortex differed from methylation in blood by more than 50% (differential (diff) methylation >50%). Positive diff methylation indicates relative hypermethylation of the brain-specific DMR relative to blood, whereas negative diff methylation indicates relative hypomethylation, with an FDR < 0.00005.*

2) The use of unmethylated BD (page 17, line 180) should be defined with further

granularity. Does this refer to unmethylated regions (UMR) defined as 0-5% methylated which typically correspond to promoter-like elements (see citation below)? When referring to “levels of unmethylated BD” (page 17, line 182), is this referring to the proportion of BD that are unmethylated?

**RESPONSE: See previous comment and response where we revised that section to also revise with this comment as well.**

3) In addition to defining BD with increased granularity, Fig C would be further clarified by a Y-axis range of 0-100%. Additionally, the figure would be clearer if the Y-axis was titled “Percentage unmethylated BD” .

**RESPONSE: We revised based on the above comment and includes this updated figure.**

*

*

4) Has the raw data been made publicly available (i.e., deposited into a public repository)?

**RESPONSE: We uploaded the raw data for all three figures to Mendeley Data**

Minor comments:
1) Throughout the manuscript, please be cautious about use of plural vs singular (e.g., has vs have).

**RESPONSE: As seen in our manuscript we revised to increase clarity of our manuscript**

2) The conclusion on page 20, line 224, “…BD, can be used to trace the efficacy of CO treatment,” may be overstated as there are no clinical correlates provided in this study. It would potentially be more accurate to note that this study demonstrated the differential methylation of BD in response to intervention. This could potentially be correlated to clinical outcomes and serve as a useful biomarker in the future.

**RESPONSE:** **We revised as recommended above**

Page 13 Paragraph 2

*“We then established a brain methylation atlas and found that brain-specific cfDNA, BD, demonstrated differential methylation in response to intervention. This could potentially be correlated to clinical outcomes and serve as a useful biomarker in the future. We demonstrated that plasma cfDNA shows great potential to identify tissue-specific secondary injuries, and this feature makes cfDNA biomarkers appealing in the field of critical care, where most biomarkers still lack tissue specificity. In sum, cfDNA biomarkers can provide critical insights into the effectiveness of therapeutic interventions, potentially guiding adjustments in clinical strategies to improve patient outcomes. “*

3) Though not necessary, it may be worthwhile to also highlight the short half-life of cfDNA which adds to its utility as a biomarker relative to other delayed biomarkers in clinical practice.

**RESPONSE: We did revise the introduction to dicuss the short half-life as recommended above**

*Introduction on page 5 last paragraph*

*“Lactate, for example, often indicates general cellular dysfunction from poor perfusion or mitochondrial injury but a limitation is that it is not clear where the lactate specifically comes from. The short half-life of cfDNA makes it an appealing tool for tracking genetic changes in real time, adding to its utility as a biomarker relative to other delayed biomarkers in clinical practice. In this protocol study, we describe the potential utilization of cfDNA as a biomarker in our swine model using carbon monoxide as a therapy in cardiac arrest that may allow other investigators to apply our method.“*
