# Supplementary material for: The application of cell-free DNA methylation patterns in critical illnesses: A protocol paper
Source: PLoS One. 2025 May 12;20(5):e0321626. doi: 10.1371/journal.pone.0321626 (PMC12068884; doi:10.1371/journal.pone.0321626)
Supplement: Attachment — Submitted filename: Response_to_Reviewers_auresp_2.docx [file pone.0321626.s003.docx]

**PONE-D-24-42835**

**The application of cell-free DNA methylation patterns in critical illnesses protocol**

**PLOS ONE**

Dear PLOS ONE,

Thank-you for the reviewer comments to improve this paper and request for minor revisions. We have submitted a second revised manuscript that should address all the feedback. Please let us know if you require any additional information and/or changes.

We have included the following for this submission:

- [X] A rebuttal letter that responds to each point raised by the academic editor and reviewer(s). You should upload this letter as a separate file labeled 'Response to Reviewers'.
- [X] A marked-up copy of your manuscript that highlights changes made to the original version. You should upload this as a separate file labeled 'Revised Manuscript with Track Changes'.
- [X] An unmarked version of your revised paper without tracked changes. You should upload this as a separate file labeled 'Manuscript'.

For this submission we also have addressed the following and/or respond to the following:

**1. General Comment: When responding to reviewer comments, the authors should improve the materials and methods sections, by properly detailing the methods, including following proper sample collection procedures. The authors should also improve the discussion of obtained results.**

*RESPONSE: We have revised the manuscript to make the recommended changes largely in response to Reviewer 3 below. As can be seen in our tracked changed manuscript that focuses on the revision of material and methods and discussion. Please see below for the specific changes.*

**2. Reviewer #1: The authors addressed all points raised and modified the manuscript accordingly. The manuscript can now be published.**

*RESPONSE: Thank-you for the positive comments and that the manuscript can now be published.*

**3. Reviewer #3: The manuscript delineates a protocol for the analysis of cell-free DNA methylome in critical care medicine. This method has been evaluated in an animal model and shows promise for translation to human applications.**

However, the manuscript's language is often cumbersome, lacking a coherent structure, and gives an overall unprofessional impression. The English language needs to be refined, and it is strongly recommended that the text undergo a thorough review and correction by a scientific editor.

*Response: We have revised the manuscript and as seen in our tracked change manuscript we have ensured enhanced readability.*

The title is misleading; it should be corrected to reflect the nature of the study.

*Response: We have changed the title to “***The application of cell-free DNA methylation patterns in critical illnesses: A protocol paper.“**

*This is in line with similar protocol publications with PLOS One such as:*

*1. An evaluation of the Live Well Erie workforce development program, jumping off the benefits cliff: A protocol paper.*

*2. Prediction of Ovarian Hyperstimulation Syndrome in Patients Treated with Corifollitropin alfa or rFSH in a GnRH Antagonist Protocol.*

1. Introduction:
The utilization of cfDNA for the purpose of tracking genetic alterations is not pertinent to the subject at hand.
The objective of the study is not adequately delineated.

*Response: Based on the above comment we removed “The short half-life of cfDNA makes it an appealing tool for tracking genetic changes in real time, adding to its utility as a biomarker relative to other delayed biomarkers in clinical practice.”*

*We also clearly stated the objective of the study as follows:*

*1. Introduction Section Last Paragraph*

*“The objective of this protocol study is to describe the potential utilization of cfDNA as a biomarker in our swine model using carbon monoxide as a therapy in cardiac arrest as an example of our method.”*

2. Abbreviations must be clarified at the first occurrence, e.g., n-cfDNA, mtDNA, etc.

*Response: We have revised to what is recommended above. For example:*

*Introduction Paragraph 2*

*“The main source of cfDNA is typically actively secreted by cells and can also be released by cells upon death. cfDNA are derived from either nuclear cfDNA (n-cfDNA) or mitochondrial cfDNA (mt-cfDNA). Early research with cfDNA was in the field of oncology to detect specific sequences or mutations to guide chemotherapy for patients as rapidly dividing cells often release cfDNA. CfDNA has also served a role in the prognosis of disease progression as well as response to treatment in other disease states. There is also controversy that cfDNA may also act as a damage-associated molecular patterns (DAMPs) that can lead to sterile inflammation. While inflammation can have positive benefits such as tissue repair in the acute phase, long-term inflammation can lead to neurogenerative diseases and autoimmune diseases. While it is possible cfDNA may directly lead to inflammation, data from supporting studies conclude that cfDNA does not directly contribute to disease progression but more likely serves as a biomarker of cellular stress or cell death.”*

3. Materials and methods:
The term "ongoing large animal study" is employed, yet it is evident that a total of four animals were utilized in the study. This inaccuracy must be rectified.

*Response: We clarified to state as an ongoing study we have completed 4 animals with 6 more animals that are planned and that we used the collected data to illustrate the methods in this paper.*

*Material and Methods*

*“In this ongoing swine study to demonstrate our protocol, all procedures were approved by the Institutional Animal Care and Use Committee at the Children’s Hospital of Philadelphia (CHOP) and performed in accordance with the National Institutes of Health Guide for the Care and Use of Laboratory Animals (Protocol 1443). As an ongoing study, we have completed four animals out of the ten animals.*

4. The procedure for blood processing is inadequately detailed, and the time frame between sample collection and centrifugation, the duration of the centrifugation process, the rationale for utilizing RT instead of 4°C, and the quantity of plasma samples utilized for cfDNA extraction should be clarified.

*Response: We revised the relevant section to include the recommendations above*

***Blood collection, cell-free DNA extraction, and bisulfite conversion***

*To illustrate our protocol levering our established swine model of cardiac arrest with the use of CO as a therapy****,*** *blood samples were then collected following a standard protocol using cfDNA/cfRNA Preservative Norgen tubes (Norgen Biotek, Canada). The following protocol is based on Norgen Biotek’s recommendation with respect to centrifugation speed and temperature. A total of 20 ml of whole blood placement into two Norgen tubes, each tube was then gently inverted 5 times to allow gentle mixture of the preservative and the blood samples followed immediately for processing. Samples were then centrifuged at 1,600 x g for 10 min at 4°C to obtain a total of 10 mL of plasma on the upper layer. For cfDNA, the plasma was then centrifuged at 16,000 x g at RT to remove cell debris. The supernatant was then stored at -80°C before cfDNA extraction. cfDNA extraction was then performed with the NextPrep-Mag cfDNA isolation kit (PerkinElmer, MA), following the manufacturer’s protocol. CfDNA quantification was performed using Qubit 1x dsDNA HS assay kit with the Qubit 4 fluorometer (Thermo Fisher Scientific). Swine cortex (PG-212) and blood (PG-705) genomic DNA (gDNA) samples were acquired from Zyagen as reference genomes. Samples were bisulfite converted using EZ DNA methylation-lightning kit (Zymo Research) before sequencing and data processing.*

5. Has it been tested that the "brain-specific" DMRs are not specific to other tissues (excluding blood)?

*Response: We appreciate the reviewer’s comment**. So far, we have methylation data from brain and blood, but not from other tissues. Therefore, we cannot determine whether the brain-specific DMRs are exclusive to the brain. It would be of great interest to collect other tissues and investigate secondary damage in cardiac arrest.*

6. The Results section is inadequately structured and not sufficiently explained. Please share more details.

*Response: We revised the results section with the above feedback*

*Result*

*We found that cfDNA methylation patterns were distinctively clustered at 2h post CO treatment and at the end of the observation andthat the cfDNA components varied between the two groups [****Fig A****].. The blood tissue served as a background tissue as most of the cfDNA fragments in plasma are derived from the apoptotic hematopoietic cells in healthy subjects. A group of hypermethylated brain DMRs (in red) and a group of hypomethylated brain DMRs (in blue) [****Fig B****]. Then a comparison of the cfDNA methylome to the methylation atlas and we found a hypomethylated brain DMR (BD). The level of unmethylated BD, which is the proportion of BD that is not methylated, significantly increased at 2h post sham treatment and at the end of observation [****Fig. C****]. On the contrary, the levels of unmethylated BD were kept constant during and after CO treatment*

7. The discussion section is similarly deficient in clarity and depth. References should be added to support the statements made in the discussion section.

*Response: We clarified and revised the entire discussion with the appropriate references.*

*Discussion*

*To examine whether CO treatment makes a difference to animals suffering from cardiac arrest, we profiled the cfDNA methylome in Sham and CO groups using two animals in each group to illustrate our methods.*

*To achieve this, we combined our expertise in cardiac arrest and CO poisoning as a therapy in cardiac arrest to primarily illustrate the potential application of cfDNA as an innovative biomarker. In our study we demonstrate the protocol and method to use cfDNA to help localize tissue source using our swine model of acute injury.^12,13^*

*One of the strengths of cfDNA when compared to other biomarkers include the ability to use bulk concentrations of cfDNA to correlate with severity of disease illness such as the use of lactate in sepsis.^5^ For instance, the degree of lactate elevation can correlate with degree of tissue hypoperfusion in a wide range of cellular hypoxia. For example, the degree of lactate elevation can correlate with the degree of cyanide poisoning. Another example where biomarker levels are used is the use of cholinesterase activity to gauge the severity of organophosphate (OP) poisoning.^3,16^ As with many clinically used biomarkers, cholinesterase activity has poor correlation with severity. We compared the use of cfDNA against cholinesterase activity in a rodent model of chlorpyrifos and found that cfDNA was a more sensitive biomarker of OP poisoning.^13^*

*Another advantage that cfDNA has to offer in addition to measuring cfDNA concentrations is also the ability to provide the tissue origin that may localize the site of injury. Others have leveraged the use of cfDNA to provide tissue localization information. One method that others have investigated is to measure the methylation pattern of cfDNA that may provide the tissue source of the DNA. Methylation of cytosine adjacent to guanine (CpG) is an essential component of cell-specific gene regulation and serves as a marker of cell identity. cfDNA molecules from loci carrying tissue-specific methylation can be used to identify cell death in a specific tissue.^17^ Tissue-specific methylation on cfDNA can be potentially used to identify brain cell death (from other sources such as blood cells) and to evaluate post-injury neuro-recovery in the brain and central nervous system in future studies.*

*CfDNA has been shown to be useful biomarkers in diseases such as traumatic brain injury and cancer.^8,9,18^ Here, we want to explore the possibility of utilizing cfDNA in monitoring other secondary organ injuries caused by cardiac arrest. We showcased a proof-of-concept brain-specific cfDNA biomarker in our data. We first found that the cfDNA methylation profiles were different between groups.* *We then established a brain methylation atlas and found that brain-specific cfDNA, BD, demonstrated differential methylation in response to intervention. This could potentially be correlated to clinical outcomes and serve as a useful biomarker in the future. We demonstrated that plasma cfDNA shows great potential to identify tissue-specific secondary injuries, and this feature makes cfDNA biomarkers appealing in the field of critical care, where most biomarkers still lack tissue specificity.*

*Since our interest was to illustrate cfDNA as a biomarker to gauge the secondary organ injury, particularly the brain damage, we built the swine brain methylation atlas using genomic DNA isolated from the swine brain and blood tissue. These data suggest that CO treatment might protect the brain from being further injured during cardiac arrest. In sum, brain-specific cfDNA can potentially serve as a biomarker to monitor the progression of the injury and the response to treatment. In conclusion, cfDNA biomarkers can provide critical insights into the effectiveness of therapeutic interventions, potentially guiding adjustments in clinical strategies to improve patient outcomes.*

***Limitations***

*While this is primarily a protocol paper for application in other similar animal models of critical illnesses there are some limitations to be aware of. While we highlighted the differences in the two groups looking at the DNA methylation patterns, the results may vary depending on the injury model. This protocol study provides proof of concept for the utility of cfDNA to study tissue involvement in critical illnesses that may help to guide management. Another limitation is now the time, and cost may be prohibitive in the acute phase of cardiac arrest but can have potential application in the post-arrest phase once the patient has been stabilized and prognostics tests are being performed. Finally, another current limitation is that . So far, we have methylation data from brain and blood, but not from other* tissues*. Therefore, we cannot determine whether the brain-specific DMRs are exclusive to the brain. It would be of great interest to collect other tissues and investigate secondary damage in cardiac arrest.*
